# RNA Folding and Catalysis Mediated by Iron (II)

**DOI:** 10.1371/journal.pone.0038024

**Published:** 2012-05-31

**Authors:** Shreyas S. Athavale, Anton S. Petrov, Chiaolong Hsiao, Derrick Watkins, Caitlin D. Prickett, J. Jared Gossett, Lively Lie, Jessica C. Bowman, Eric O'Neill, Chad R. Bernier, Nicholas V. Hud, Roger M. Wartell, Stephen C. Harvey, Loren Dean Williams

**Affiliations:** 1 School of Biology, Georgia Institute of Technology, Atlanta, Georgia, United States of America; 2 School of Chemistry and Biochemistry, Georgia Institute of Technology, Atlanta, Georgia, United States of America; 3 NAI Center for Ribosomal Origins and Evolution, Georgia Institute of Technology, Atlanta, Georgia, United States of America; University of Florida, United States of America

## Abstract

Mg^2+^ shares a distinctive relationship with RNA, playing important and specific roles in the folding and function of essentially all large RNAs. Here we use theory and experiment to evaluate Fe^2+^ in the absence of free oxygen as a replacement for Mg^2+^ in RNA folding and catalysis. We describe both quantum mechanical calculations and experiments that suggest that the roles of Mg^2+^ in RNA folding and function can indeed be served by Fe^2+^. The results of quantum mechanical calculations show that the geometry of coordination of Fe^2+^ by RNA phosphates is similar to that of Mg^2+^. Chemical footprinting experiments suggest that the conformation of the *Tetrahymena thermophila* Group I intron P4–P6 domain RNA is conserved between complexes with Fe^2+^ or Mg^2+^. The catalytic activities of both the L1 ribozyme ligase, obtained previously by *in vitro* selection in the presence of Mg^2+^, and the hammerhead ribozyme are enhanced in the presence of Fe^2+^ compared to Mg^2+^. All chemical footprinting and ribozyme assays in the presence of Fe^2+^ were performed under anaerobic conditions. The primary motivation of this work is to understand RNA in plausible early earth conditions. Life originated during the early Archean Eon, characterized by a non-oxidative atmosphere and abundant soluble Fe^2+^. The combined biochemical and paleogeological data are consistent with a role for Fe^2+^ in an RNA World. RNA and Fe^2+^ could, in principle, support an array of RNA structures and catalytic functions more diverse than RNA with Mg^2+^ alone.

## Introduction

When large RNAs fold into compact structures, negatively charged phosphate groups achieve close proximity. Folded RNAs are stabilized in part by inorganic cations that accumulate in and around the RNA envelope. ‘Diffuse’ cations remain hydrated and make primary contributions to global stability by mitigating electrostatic repulsion of the negatively charged backbone. Chelated ions are less frequent, but in some instances are essential for achieving specific local conformation of the RNA. A special importance of Mg^2+^ in tRNA folding was seen early on [1]–[3]. It is now known that Mg^2+^ plays important roles in folding of essentially all large RNAs [4]–[6]. In addition, Mg^2+^ ions assist directly in stabilizing transition states of some ribozymes [7], [8].

Here we use computation and experiment to address the question of whether Fe^2+^ can substitute for Mg^2+^ in RNA folding and catalysis. Mg^2+^ possesses important electronic and geometric properties that are key to its relationships with RNA. It is redox inactive, and does not cleave RNA via Fenton chemistry. The ionic radius of Mg^2+^ is small, the charge density is high, the coordination geometry is strictly octahedral, and the hydration enthalpy is large and negative [9]–[11]. In comparison with Group I cations, calcium, or polyamines, Mg^2+^ has a greater affinity for phosphate oxygens [12]. We find that Fe^2+^ is an excellent Mg^2+^ mimic in the absence of O_2_, readily substituting for Mg^2+^ in RNA folding and catalysis.

Our primary motivation is to study RNA under plausible early earth conditions. Understanding the influence of Fe^2+^ on RNA structure and function could provide important links between the geological record and the RNA world. It is believed that life originated with RNA-based genetic and metabolic systems, i.e. the RNA world [13], which apparently flourished in an anoxic environment in which iron was much more soluble and abundant than in our current oxidative environment. Life evolved for around a billion years before the rise of photosynthesis and the Great Oxidation Event [14], [15]. Fe^2+^, either instead of or in combination with Mg^2+^, seems to be a possible partner of RNA in the biology of the pre-photosynthesis anoxic earth.

With the rise in free oxygen, a product of photosynthesis, the Fe^2+^ of the early earth was oxidized and sequestered. Iron was deposited in banded iron formations (BIFs) [16], but BIF iron is seen by isotopic variations to have been a participant in ancient biological processes [17]. The transition from soluble to insoluble iron caused slow but dramatic shifts in biology and geology.

## Results

### Theory predicts that RNA conformation is conserved if Fe^2+^ substitutes for site-bound Mg^2+^

**Figure 1:**
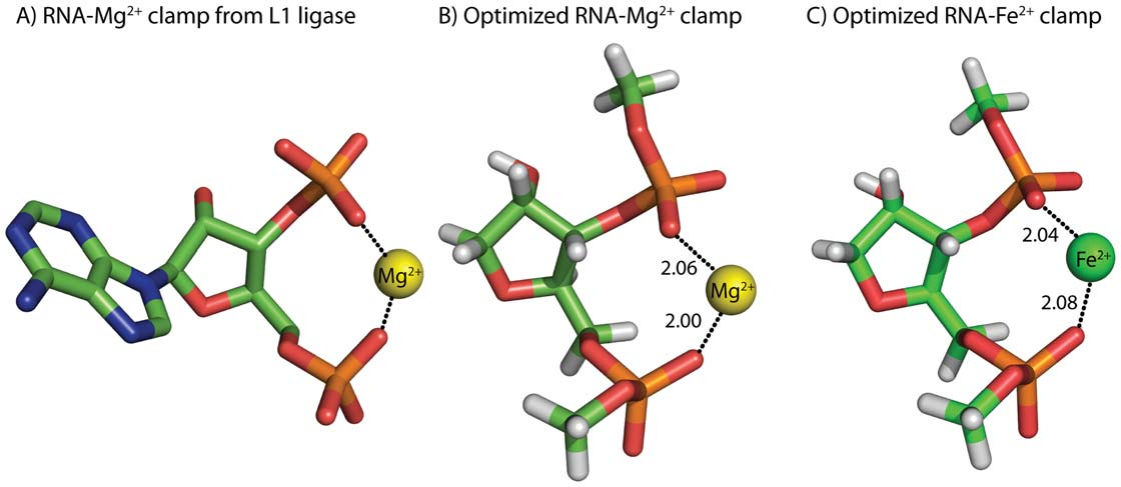
Conformations of RNA-Mg^2+^ and RNA-Fe^2+^ clamps are nearly identical. A) RNA-Mg^2+^ clamp from the L1 ribozyme ligase (PDB 2OIU). B) RNA-Mg^2+^ clamp optimized by high level QM calculations. C) An optimized RNA-Fe^2+^ clamp. Each cation (Mg^2+^ or Fe^2+^) is hexacoordinate. Mg^2+^ is shown as a yellow sphere and Fe^2+^ is shown as a green sphere. Water molecules are omitted from the images for clarity. Distances are in Å.

Quantum mechanical (QM) calculations show that RNA conformation and coordination geometry are conserved when Mg^2+^ is replaced by Fe^2+^ in first shell RNA-metal complexes. We focused here on an RNA-Mg^2+^ clamp [18], in which two adjacent RNA phosphates coordinate a common Mg^2+^ (Figure 1A). A complex with multiple first-shell RNA interactions with Mg^2+^ should provide a stringent test of the ability of Fe^2+^ to substitute. RNA-Mg^2+^ clamps are common in large RNAs [19]. One observes twenty-five RNA-Mg^2+^ clamps in the *Haloarcula marisortui* large ribosomal subunit [20], two in the P4-P6 domain of the *Tetrahymena thermophila* Group 1 intron [21], one in a self-splicing group II intron from *Oceanobacillus iheyensis* [22], and one in the L1 ribozyme ligase [23]. The folding and function of each of these RNAs is Mg^2+^ dependent.

The conformations of an RNA-Mg^2+^ clamp and an RNA-Fe^2+^ clamp are nearly identical, as determined by Density Functional Theory (DFT) [24]. The RNA conformation and metal-oxygen distances and angles are very similar (Figures 1B and 1C). The calculations do indicate some subtle differences, however. Calculated interaction energies (energies of complex formation, Table S1) favor Fe^2+^ over Mg^2+^ by 1.3 kcal/mol in continuum solvent as indicated by DFT calculations. Natural Bond Orbital analysis [25] in the gas phase (Table S2) suggests that more charge is transferred from phosphate to Fe^2+^ (0.43 e-) than from phosphate to Mg^2+^ (0.29 e-). This difference implies that compared to Mg^2+^, Fe^2+^ better activates the phosphorous of RNA to nucleophilic attack. The increase in activation is attributable to the accessibility of the d-orbitals of Fe^2+^.

### Chemical probing suggests that RNA conformation is conserved when Fe^2+^ substitutes for Mg^2+^

Selective 2′-hydroxyl acylation analyzed by primer extension (SHAPE) is a powerful RNA foot-printing technique that provides structural information at single-nucleotide resolution [26]–[28]. SHAPE has been used to accurately predict and confirm secondary structures of RNA ranging in length from tRNA to the HIV-1 genome [27], [29]. The method exploits the reactivity of the 2′-hydroxyl groups of RNA to electrophiles to form 2′-O-ribose adducts. Here we employed the SHAPE reagent benzoyl cyanide (BzCN). The relative reactivities of ribose 2′-hydroxyl groups to the electrophile are sensitive to local RNA flexibility, which depends primarily on whether or not a nucleotide is base-paired. Single-stranded nucleotides react preferentially over double-stranded nucleotides. Reverse transcription using fluorescently labeled primers gives products that are truncated at locations indicating 2′-O-ribose adducts. The fragments are resolved and visualized using capillary electrophoresis. Capillary electrophoresis data were processed as described [30].

**Figure 2:**
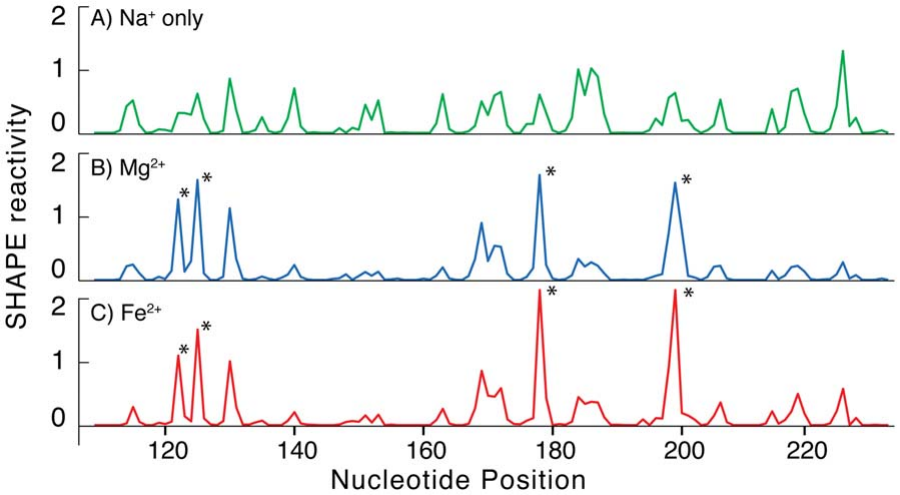
Addition of Mg^2+^ or Fe^2+^ causes the same changes in the SHAPE reactivity of the P4–P6 domain of the *T. thermophila* Group 1 intron. A) Shape profile in presence of 250 mM NaCl and no divalent cations. B) The addition of Mg^2+^ increases the reactivity at the sites indicated with the asterisks and decreases reactivity at other sites. This reaction contains 2.5 mM Mg^2+^ and 250 mM NaCl. C) The addition of Fe^2+^ causes the same changes in SHAPE reactivity as Mg^2+^. This reaction contains 2.5 mM Fe^2+^ and 250 mM NaCl.

The secondary structure of the *T. thermophila* Group I intron P4–P6 domain was assayed by SHAPE in the presence of Na^+^ alone, giving a reaction pattern consistent with the known secondary structure [21] (Figure 2A). For example, in the stem-loop formed by residues 143–160, the double-stranded nucleotides of the stem are unreactive while the GAAA nucleotides of the loop are reactive. Some of the most reactive nucleotides of the P4–P6 domain secondary structure are located within the A-rich bulge (nucleotides 182–188).

We probed the structure of the P4–P6 domain RNA in presence of Mg^2+^ (Figure 2B). The folding of RNAs from secondary structure to compact native states, containing long-range tertiary interactions, is known to be Mg^2+^-dependent [4]–[6]. The addition of 2.5 mM Mg^2+^ to the P4–P6 domain RNA causes pronounced changes in the SHAPE reactivity. SHAPE reactivity increases at nucleotides 122, 125, 177–179 and 198–200 (indicated by asterisks in Figure 2B). The Mg^2+^-dependence of SHAPE reactivities reflects (i) specific magnesium binding, (ii) diffuse interactions of Mg^2+^, and (iii) RNA-RNA tertiary interactions facilitated by Mg^2+^, as previously demonstrated for tRNA [27], RNase P [31], and Domain III of the ribosomal large subunit [30]. The pattern of SHAPE reactivity for P4–P6 domain RNA in the presence of Mg^2+^ observed here is nearly identical to that described previously for the same RNA in the presence of Mg^2+^ by Cech and coworkers (using N-methylisatoic anhydride instead of BzCN) [32].

The pattern of SHAPE reactivity for P4–P6 domain RNA is conserved when Mg^2+^ is replaced by Fe^2+^ under anaerobic conditions. Figure 2C shows that SHAPE reactivities in presence of 2.5 mM Fe^2+^ are identical, within the accuracy of the experiment, to those in presence of 2.5 mM Mg^2+^. These results suggest that tertiary interactions and even the ‘ion core’ of the P4–P6 domain are recapitulated by Fe^2+^ in the absence of oxygen. As expected, if Fe^2+^ is added to the RNA in the presence of atmospheric free oxygen, the RNA is quickly degraded (not shown).

### Activity of two ribozymes is enhanced by Fe^2+^ compared to Mg^2+^

**Figure 3:**
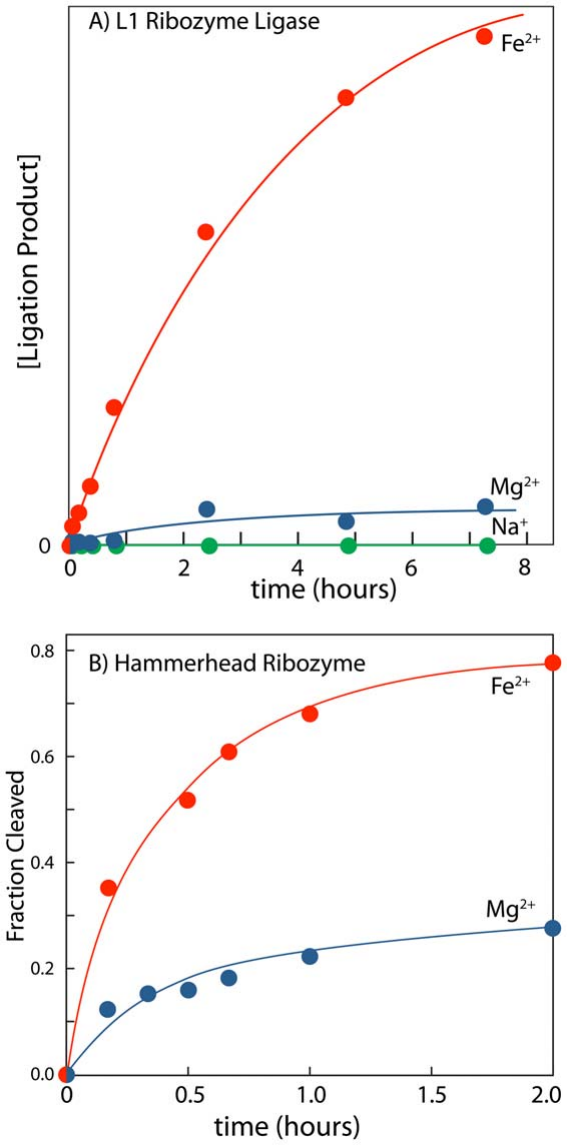
Ribozyme activity is enhanced by Fe^2+^ compared to Mg^2+^. A) L1 ribozyme ligase activity is enhanced in Fe^2+^ compared to Mg^2+^. Ligase reactions were performed under anaerobic conditions at room temperature and 250 mM Na^+^ in 100 µM [Fe^2+^] or 100 µM [Mg^2+^]. The reaction components were first annealed in 50 mM HEPES, pH 8.0, 200 mM sodium acetate by incubating at 90°C for 3 min and cooling to room temperature over 30 min. The ligation reaction was initiated by adding the appropriate cation salt. The Na^+^ only reaction gave no product. Reaction progress was monitored by gel electrophoresis. B) Hammerhead ribozyme activity is enhanced in Fe^2+^ compared to Mg^2+^. Hammerhead ribozyme cleavage reactions were performed under anaerobic conditions at room temperature in 50 mM HEPES, pH 7.5 and 25 µM [Fe^2+^] or 25 µM [Mg^2+^]. Substrate and ribozyme RNA strands were first annealed in 50 mM HEPES buffer by incubating at 90°C for 2 min and cooling to room temperature over 30 min. Cleavage reactions were initiated by addition of FeCl_2_ or MgCl_2_ from stock solutions. Reactions were monitored by both gel electrophoresis and capillary electrophoresis, which gave similar results.

To investigate RNA function in presence of Fe^2+^, we tested the catalytic activity of the L1 ribozyme ligase in the presence of Mg^2+^ or Fe^2+^ (in the absence of oxygen). This ligase catalyzes formation of a phosphodiester linkage. The 3′-hydroxyl group of an RNA substrate attacks the α-phosphorus of the ribozyme 5′-triphosphate [23]. This ribozyme was selected *in vitro* in the presence of high [Mg^2+^] (60 mM) by Robertson and Ellington, and has been described as Mg^2+^-dependent [23]. The initial rate of ligation in 100 µM Mg^2+^ is 1.4×10^−6^ min^−1^, while the initial rate of ligation in 100 µM Fe^2+^ is 3.5×10^−5^ min^−1^, which is 25-fold higher (Figure 3A). A higher rate for Fe^2+^ over Mg^2+^ holds for essentially any reasonable equimolar concentration of the two cations. Achieving an equivalent rate of reaction requires around a 10-fold greater [Mg^2+^] than [Fe^2+^]. As expected, this ligase is inactive in Na^+^ alone. This control, along with the chemical footprinting of the P4–P6 domain in Na^+^ alone (Figure 2A), confirms the efficacy of our divalent cation extraction procedure using divalent cation chelating beads.

The hammerhead ribozyme was also assayed for activity in the presence of Fe^2+^. Hammerhead ribozyme sequences are widely distributed in the tree of life [33]. This ribozyme cleaves the RNA backbone via nucleophilic attack by a 2′-hydroxyl group on the 3′-phosphorous atom [34]. In these reactions the initial rate of hammerhead cleavage in 25 µM Mg^2+^ is 0.011 min^−1^, while the initial rate of cleavage in 25 µM Fe^2+^ is 0.035 min^−1^, which is 3-fold higher (Figure 3B). The maximum fraction of cleaved substrate was about 3-fold greater in Fe^2+^ versus Mg^2+^. When 100 µM of these two divalent cations were employed, Fe^2+^ again showed a higher initial rate of cleavage of ∼3.5 fold (data not shown).

## Discussion

The results here support a model of early evolution in which Fe^2+^ was an important metallo-cofactor for RNA. In this model, Fe^2+^ was replaced over time by Mg^2+^, by processes driven at least in part by photosynthesis and the Great Oxidation Event.

### The RNA World – on Steroids

The RNA world is hypothesized to have occurred during the early Archean eon, prior to the Great Oxidation Event. Fe^2+^ in the early Archean would have been available, soluble and non-toxic. Our observations here of Fe^2+^-mediated RNA folding and catalysis, in combination with paleogeological information, suggest that RNA could have originated and evolved in association with Fe^2+^. The RNA-Fe^2+^ complexes recently observed in extant biology [35] could be molecular fossils from the RNA world, akin to the ribosome. The injection of Fe^2+^ into RNA World models opens broad new possibilities for ancient biochemistry. RNA and Fe^2+^ could, in principle, support an array of RNA structures and catalytic functions far more diverse than RNA with Mg^2+^ alone. Complexes of RNA with Fe^2+^ offer the prospect of redox chemistry and electron transfer reactions for ancient ribozymes.

### Replacement of Fe^2+^ by Mg^2+^ in RNA is analogous to replacement of Fe^2+^ by Mn^2+^ in protein enzymes

The conversion of one metal to another is facile in some protein enzymes. In just one example, Mn^2+^ and Fe^2+^ are used as cofactors in a broad class of superoxide dismutases (Fe^2+^/Mn^2+^ SODs) [36]. The metal cofactors of these SODs can be interconverted between Mn^2+^ and Fe^2+^ while the coordination geometry, amino acid sequence and global fold of the protein are conserved. The discrimination between Fe^2+^ and Mn^2+^ in Fe^2+^/Mn^2+^ SODs *in vivo* is determined by species, organelle, protein isozyme, protein expression level and metal bioavailability. Metal substitution appears to be a useful biological strategy in nutrient-limited environments [37]–[39]. Falkowski has proposed that during the Great Oxidation Event, Mn^2+^ was appropriated into some Fe^2+^ dependent enzymes [37]. Here we suggest that the same strategy was employed with RNA, where Fe^2+^ was converted to Mg^2+^. It has been suggested, based on sites of Fenton cleavage (using iron/O_2_), that Fe^2+^ and Mg^2+^ compete for common sites in RNA [40]–[42] *in vitro*.

### QM calculations suggest that an RNA fragment that forms multiple first-shell interactions with Mg^2+^ does not change conformation when Mg^2^ is replaced by Fe^2+^

The metal-oxygen distances and angles are nearly identical in the Mg^2+^ and Fe^2+^ complexes (Figures 1B and 1C). The QM calculations do indicate subtle differences between Mg^2+^ and Fe^2+^ complexes. It appears that more charge is transferred from phosphate to Fe^2+^ than from phosphate to Mg^2+^. This electronic difference, which activates the phosphorus atom to nucleophilic attack, is attributable to the accessibility of the d-orbitals of Fe^2+^.

### Chemical probing experiments in solution, using the T. thermophila Group I intron P4–P6 domain RNA, demonstrate the ability of Fe^2+^ to substitute for Mg^2+^ during folding of large RNAs

The P4–P6 domain interacts with Mg^2+^ by a complex blend of diffuse and chelated modes [21], [43], [44]. In spite of this complexity, the changes in SHAPE reactivity of RNA induced by association with Mg^2+^ or Fe^2+^ in the absence of free oxygen are very similar (Figures 2B and 2C). SHAPE reports local RNA flexibility, which depends primarily on whether or not a nucleotide is base-paired in secondary or tertiary interactions. The results for the P4–P6 domain suggest that in the absence of free oxygen, Fe^2+^ can replace Mg^2+^ in compacting and folding large RNAs. Thus it appears that Fe^2+^ and Mg^2+^ are nearly interchangeable in their interactions with RNA.

### Fe^2+^ can substitute for Mg^2+^ to support catalysis by ribozymes

At equimolar concentrations of Mg^2+^ or Fe^2+^, the initial rate of ligation observed for the L1 ribozyme ligase is 25-fold higher with Fe^2+^ than Mg^2+^ in the absence of free oxygen (Figure 3A). Similarly, at equimolar concentrations of Mg^2+^ or Fe^2+^, the initial rate of RNA cleavage observed for the hammerhead ribozyme is 3-fold higher with Fe^2+^ than Mg^2+^ (Figure 3B). In sum, we have looked at RNA folding in three independent experimental systems, by chemical footprinting (P4–P6 domain), and with two ribozyme assays (L1 ribozyme ligase and the hammerhead ribozyme). In each system examined, Fe^2+^ substitutes for Mg^2+^ in the absence of free oxygen. The increased activities of the ribozymes with Fe^2+^ over Mg^2+^ are consistent with our computational results that suggest Fe^2+^ is slightly better than Mg^2+^ at activating the phosphorous of RNA to nucleophilic attack. In the hammerhead, which is one of the best-characterized ribozymes, it has been shown that a Mg^2+^ ion interacts directly with the scissile phosphate before and during catalysis [45]. The results here suggest that Fe^2+^ is a superior substitute for Mg^2+^ in this catalytic role. A variety of effects such as differential affinity of Fe^2+^ and Mg^2+^ globally, or for various sites on RNA, could also contribute to differences in cleavage rates.

### How much Fe^2+^ was available during the time of the RNA World?

It seems very likely that the early Archean earth provided a variety of Fe^2+^-rich microenvironments. On a global scale, the [Fe^2+^]_marine_ in the early Archean is subject to debate, and is largely circumscribed in current models by P_CO2_ (atmospheric pressure of CO_2_) in the atmosphere (Fe^2+^ precipitates as siderite: Fe^2+^CO_3_^−^). Early earth P_CO2_ is inferred using estimates of greenhouse effects, sun luminosity, earth albedo and temperatures required to maintain liquid oceans. A variety of recent results challenge high P_CO2_ models [46], [47]. If P_CO2_ was low, [Fe^2+^]_marine_ could have been as high as 100–1000 µM [14], [48], compared to 0.3–0.8 nM in the modern ocean [49]. P_HS_ (atmospheric pressure of HS) would also have been an important influence on [Fe^2+^]_marine_ due to precipitation of FeS [50].

### How much Mg^2+^ was available during the RNA World?

The [Mg^2+^]_marine_ in the early Archean is also uncertain. Although the models are tentative, it has been suggested that [Mg^2+^]_marine_ during this time was attenuated by submarine hydrothermal systems associated with higher heat flow [51], [52], more vigorous seafloor spreading [53], [54], and by reduced Mg^2+^ delivery to the oceans by smaller continental landmasses or from the weathering of peridotite in the sea floor [55]. Both of these phenomena would tend to lower [Mg^+2^]_marine_ of early oceans in comparison to today.

### Fe^2+^ is the double-edged sword

The early earth's abundant Fe^2+^ has been oxidized and sequestered to the extent that current biomass and species diversity in many ecosystems is limited by Fe^2+^ availability [56], [57]. Iron in the presence of oxygen is rare, toxic, and biologically expensive to manage [58], [59]. Yet living systems are dependent on and must acquire and utilize iron. The concentration of iron in cells is on the order of 100 µM, with iron largely constrained to heme, iron-sulfur clusters, and di-iron or mono-iron centers, transporters, carriers, exporters, and concentrators such as ferritins [60]. Because the solubility of ferric iron in water or plasma is so low (10^−18^ M), cells must combat a massive concentration gradient. The transition from benign and abundant iron to scarce and toxic iron would have caused a slow but dramatic shift in biology that required transformations in biochemical mechanisms and metabolic pathways.

## Materials and Methods

### QM calculations

The initial atomic coordinates of a Mg^2+^-RNA clamp were extracted from the X-ray structure of the *H. marismortui* large ribosomal subunit (PDB entry 1JJ2) [20] as previously described [19]. The 5′ and 3′ phosphates were capped with methyl groups in lieu of the remainder of the RNA polymer and hydrogen atoms were added, where appropriate. The Fe^2+^-RNA clamp was constructed by replacing the magnesium ion with an iron as described [18].

The binding of a Mg^2+^ or Fe^2+^ ion to an RNA fragment was described by the following reactions:

RNA^2-^ + Me^2+^(H_2_O)_6_ → RNA^2-^-Me^2+.^•(H_2_O)_4_ complex + 2H_2_O,

where Me^2+^  =  (Mg^2+^, Fe^2+^)

The reactants and products were fully optimized using density functional theory (DFT) at the B3LYP level, which combines the GGA exchange three-parameter hybrid functional developed by Becke [61] and the correlation functional of Lee-Yang-Parr [62] and the 6–311G(d,p)^++^ basis set and multiplicity = 1 as implemented in Gaussian 09 [63]. The Fe^2+^-RNA clamp and the Fe^2+^(H_2_O)_6_ were optimized at the unrestricted B3LYP/6–31G(d,p) level of theory with spin of iron = 2 and multiplicity = 5. Single point energies for these complexes were further obtained at the UB3LYP/6–311++G(d,p) level of theory using SCF options DIIS, NOVARACC, VTL, MaxCyc = 1000.

The interaction energies were calculated in water using the gas phase optimized geometries within the framework of the Integral Equation Formalism of Polarized Continuum Model [24]. The basis set superposition error (BSSE) in the dimer-centered basis set was obtained by applying the counterpoise procedure of Boys and Bernardi [64]. The corrected interaction energies were calculated by taking into account deformational energies of monomers according to the scheme proposed by van Duijneveldt-van de Rijdt and van Duijneveldt [65]. The IEFPCM approach was used to account for the effect of a polar solvent.

Natural Bond Order (NBO) [25] and Natural Energy Decomposition Analysis (NEDA) [66], [67] calculations were performed on the optimized complexes at the (U)B3LYP/6–31G(d,p) level of theory using the GAMESS package [68].

### DNA and RNA synthesis

The genes and RNA transcripts for the L1 ribozyme ligase and the P4–P6 domain the *Tetrahymena thermophila* Group 1 intron were synthesized and purified as described in Text S1. After transcription, Mg^2+^ was removed from the RNA by heat annealing in the presence of divalent cation chelating beads (Hampton Research). SHAPE reactivity and ribozyme reactions confirm that the divalent cations are removed by chelating beads.

### SHAPE probing of P4–P6 RNA

All manipulations of RNA with Fe^2+^ were conducted in a Coy chamber with an atmosphere of 85% N_2_, 10% CO_2_, 5% H_2_. P4–P6 domain RNA (11.25 µg) was lyophilized, transferred to the anaerobic chamber, left open for several hours, and resuspended in 240 µL of 50 mM HEPES, pH 8.0, 200 mM sodium acetate (final concentration) that had been previously deoxygenated by bubbling with argon for several hours. The RNA was denatured and renatured using a thermal cycler, by heating to 90°C for 3 min and then quickly cooling to 4°C. Eighty µL of the RNA solution was aliquoted into three tubes. To the first tube, 10 µL of 25 mM FeCl_2_ (Avantor Performance Materials) solution was added. To the second tube, 10 µL of 25 mM MgCl_2_ was added. To the third tube, 10 µL water was added. The tubes were incubated at room temperature for 5 minutes. The RNA from each tube was then divided equally between two additional tubes. To one tube of each pair, 5 µL of 800 mM benzoyl cyanide (BzCN) in anhydrous DMSO was added. The other tube of the pair served as a negative control, to which 5 µL neat DMSO was added. The benzoyl cyanide reactions are complete in a few seconds at room temperature [31]. The Fe^2+^ was removed by treatment with chelating beads. The beads and the associated divalent cations were removed with a 0.2 micron filtration spin column. Denaturing SHAPE experiments were performed in 20 mM HEPES pH 8.0 (final concentration) for 4 min at 90°C using 130 mM *N*-methylisatoic anhydride (NMIA) in anhydrous DMSO. Modified RNA was purified using RNeasy Mini Kit (Qiagen) and re-suspended in 20 µL 1× TE. The recovery after purification was 65–75%.

A 20-nt long DNA oligomer, 5′- GAACTGCATCCATATCAACA -3′, that anneals to the 3′-end of the P4-P6 domain, was used to prime reverse transcription. The primer was labeled with 6-FAM at its 5′-end (Eurofins MWG Operon). Reverse transcription, capillary electrophoresis and data processing were performed as described [30].

### L1 ribozyme ligation reactions

As noted above, all manipulations in which RNA was in contact with Fe^2+^ were conducted in a Coy chamber. The substrate RNA (5′-UGCACU-3′) labeled with Cy3 at its 5′-end and DNA enhancer (5′-GCGACTGGACATCACGAG-3′) were purchased from Eurofins MWG Operon. Aliquoted reaction components (L1 ligase RNA, substrate RNA and DNA enhancer; typical molar ratio used was 1∶0.1∶2, respectively) were lyophilized separately, transferred to the anaerobic chamber, left open for several hours, and resuspended in 50 mM HEPES, pH 8.0, 200 mM sodium acetate that had been previously deoxygenated by bubbling with argon for several hours. The reaction components were annealed inside the anaerobic chamber by incubating at 90°C for 3 min and cooling to room temperature over 30 min. FeCl_2_ was weighed and dissolved in water inside the anaerobic chamber. The final volumes of the reaction mixtures were generally 270 µL.

Ligation reactions were initiated by addition of the appropriate cation salt to the dissolved reaction components. At predetermined time points, 30 µL aliquots were withdrawn and quenched by treatment with chelating beads. The beads and divalent cations were removed with a spin column and the samples were frozen and stored at −80°C. The L1 ligase RNA was stable for days in 10 mM Fe^2+^ in the anaerobic chamber, but degraded quickly upon exposure to atmospheric oxygen. After the Fe^2+^ was removed with chelating beads, the L1 ligase RNA was stable to exposure to atmospheric oxygen.

For gel analysis, 5 µL of reaction mixture was mixed with 15 µL loading buffer (8 M urea, 1× TTE, 10% glycerol) and denatured by heating to 90°C for 2 min. The reaction components were then resolved on 8% denaturing PAGE gels and visualized on a Typhoon Trio variable mode imager. Some representative gels are shown in Figure S1. The band intensities were quantified using Fujifilm MultiGauge 2.0 software.

### Hammerhead ribozyme cleavage reactions

As noted above, all manipulations of the hammerhead RNA in the presence of Fe^2+^ were carried out in a Coy chamber. The hammerhead ribozyme-substrate was based on the unmodified HHα1 RNA described by Stage-Zimmermann and Uhlenbeck [69]. A 31 nucleotide substrate strand (5′-GGCAAUCGAAACGCGAAAGCGUCUAGCGGGC-3′), labeled at the 3′-end with FAM, and the 21 nucleotide ribozyme strand (5′-CCCGCUACUGAUGAGAUUGCC-3′) were purchased from IDT. Substrate and ribozyme strands (typical molar ratio used was 1∶1000) were lyophilized separately, transferred to the anaerobic chamber, left open for several hours, and resuspended in 50 mM HEPES, pH 7.5 (pH adjusted with KOH). The buffer had previously been deoxygenated by bubbling with argon for several hours. The strands were annealed inside the anaerobic chamber by incubating at 90°C for 2 min and cooling to room temperature over 30 min.

Reactions (150 µL final volume) were initiated by addition of 1.5 µL of cation solution (Fe^2+^ or Mg^2+^). At predetermined time points, 20 µL aliquots were withdrawn and quenched by treatment with divalent cation chelating beads. The beads and the associated divalent cations were removed with a spin column, and the samples were frozen and stored at −80°C. For gel analysis, 1 µL of reaction mixture was mixed with 9 µL loading buffer (8 M urea, 1× TTE, 10% glycerol) and denatured by heating to 90°C for 2 minutes. The intact 31 nucleotide substrate and 7 nucleotide product were resolved on 15% denaturing PAGE gels and visualized on a Typhoon Trio variable mode imager, or separated by capillary electrophoresis and quantified as described previously [30].

## Supporting Information

Figure S1 **8% polyacryalamide – 8 M urea denaturing gels showing L1 Ribozyme Ligase reaction progress.** Only species tagged with 5′-Cy3 dye (substrate and product) are visible. The L1 Ribozyme Ligase is visible when the gel is stained with cyber gold or ethidium. The reaction rate increases when [Fe^2+^] is increased from 100 µM (LH panel, reaction product observable at 4 hours) to 625 µM (center panel, reaction product observable at first time point, 30 min). The rate of the reaction in 1 mM Mg^2+^ (RH panel) is roughly equivalent to that in 100 µM Fe^2+^ (LH panel).
(TIF)

Table S1 **Electronic energies, interaction energies and the corresponding counterpoise-corrected interaction energies calculated at the (U)B3LYP/6–311++G(d,p) level of theory within the framework of IEFPCM in water.**
(DOCX)

Table S2 **Electronic configurations of Mg^2+^ and Fe^2+^ in the RNA^2−^ -Mg^2+^(H_2_O)_4_ and RNA^2−^ -Fe^2+^(H_2_O)_4_ complexes as revealed by the NBO at the (U)B3LYP/6–31G(d,p) level of theory.**
(DOCX)

Text S1 **RNA Synthesis and Purification.**
(DOCX)
