# Supplementary figures and images for: RNA Folding and Catalysis Mediated by Iron (II)
Source: PLoS One. 2012 May 31;7(5):e38024. doi: 10.1371/journal.pone.0038024 (PMC3365117; doi:10.1371/journal.pone.0038024)

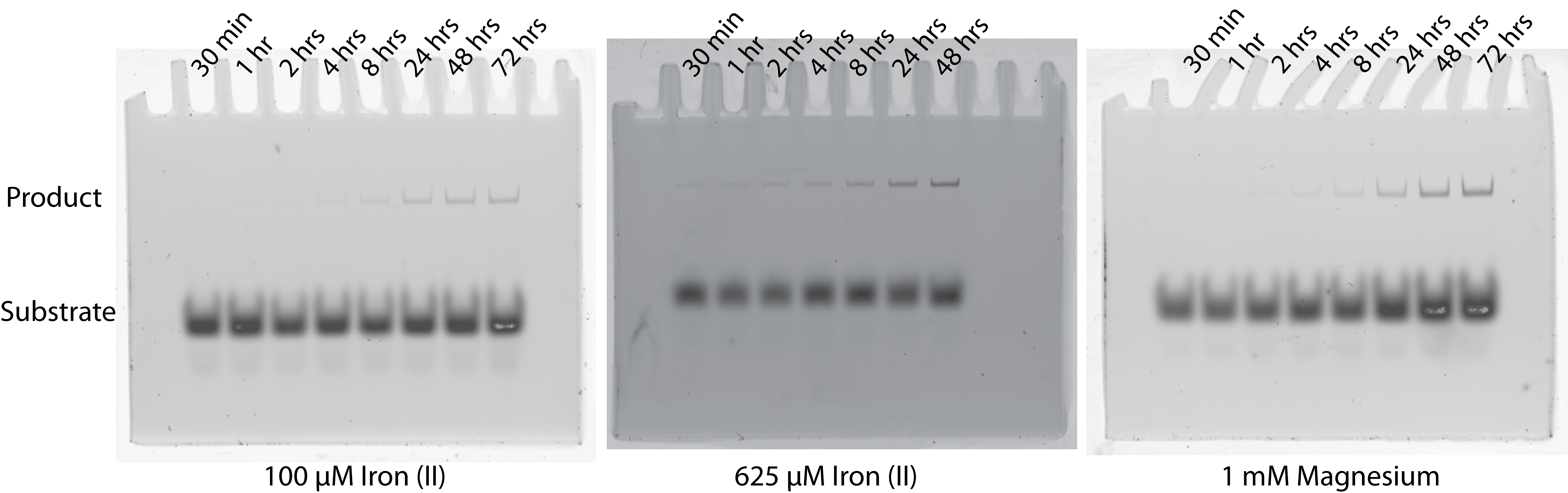

Supplement: Figure S1 — 8% polyacryalamide – 8 M urea denaturing gels showing L1 Ribozyme Ligase reaction progress. Only species tagged with 5′-Cy3 dye (substrate and product) are visible. The L1 Ribozyme Ligase is visible when the gel is stained with cyber gold or ethidium. The reaction rate increases when [Fe2+] is increased from 100 µM (LH panel, reaction product observable at 4 hours) to 625 µM (center panel, reaction product observable at first time point, 30 min). The rate of the reaction in 1 mM Mg2+ (RH panel) is roughly equivalent to that in 100 µM Fe2+ (LH panel). (TIF) [file pone.0038024.s001.tif]
