# Supplementary material for: RNA Folding and Catalysis Mediated by Iron (II)
Source: PLoS One. 2012 May 31;7(5):e38024. doi: 10.1371/journal.pone.0038024 (PMC3365117; doi:10.1371/journal.pone.0038024)
Supplement: Table S1 — Electronic energies, interaction energies and the corresponding counterpoise-corrected interaction energies calculated at the (U)B3LYP/6–311++G(d,p) level of theory within the framework of IEFPCM in water. (DOCX) [file pone.0038024.s002.docx]

Table S1. Electronic energies, interaction energies and the corresponding counterpoise-corrected interaction energies calculated at the (U)B3LYP/6-311++G(d,p) level of theory within the framework of IEFPCM in water.

| Complex | RNA^2-^ | Mg^2+^(H_2_O)_6_ | RNA^2-^-Mg^2+.^•(H_2_O)_4_ | H_2_O | IE | IE(BSSE50%)) |
| --- | --- | --- | --- | --- | --- | --- |
| RNA^2-^ -Mg^2+^(H_2_O)_4_ | -1710.95695 | -658.78488 | -2216.86735 | -76.466467 | -36.6 | -33.4 |
| RNA^2-^ -Fe^2+^(H_2_O)_4_ | -1710.95695 | -1722.29917 | -3280.38356 | -76.466467 | -37.9 | -34.7 |
