# Supplementary material for: RNA Folding and Catalysis Mediated by Iron (II)
Source: PLoS One. 2012 May 31;7(5):e38024. doi: 10.1371/journal.pone.0038024 (PMC3365117; doi:10.1371/journal.pone.0038024)
Supplement: Table S2 — Electronic configurations of Mg2+ and Fe2+ in the RNA2− -Mg2+(H2O)4 and RNA2− -Fe2+(H2O)4 complexes as revealed by the NBO at the (U)B3LYP/6–31G(d,p) level of theory. (DOCX) [file pone.0038024.s003.docx]

Table S2. Electronic configurations of Mg^2+^ and Fe^2+^ in the RNA^2-^ -Mg^2+^(H_2_O)_4_ and RNA^2-^ -Fe^2+^(H_2_O)_4_ complexes as revealed by the NBO at the (U)B3LYP/6-31G(d,p) level of theory.

| Complex | Charge | Electronic configuration |
| --- | --- | --- |
| RNA^2-^ -Mg^2+^(H_2_O)_4_ | 1.705 | [core]3s(0.26)3p(0.02)3d(0.01)4p(0.01) |
| RNA^2-^ -Fe^2+^(H_2_O)_4_ (Total) | 1.562 | [core]4s(0.29)3d(6.11)4p(0.02)5p(0.01) |
| RNA^2-^ -Fe^2+^(H_2_O)_4_ (Alpha) | -1.139 | [core]4s(0.15)3d(4.98)4p(0.01) |
| RNA^2-^ -Fe^2+^(H_2_O)_4_ (Beta) | 2.702 | [core]4s(0.14)3d(1.14)4p(0.01) |
