# Supplementary material for: RNA Folding and Catalysis Mediated by Iron (II)
Source: PLoS One. 2012 May 31;7(5):e38024. doi: 10.1371/journal.pone.0038024 (PMC3365117; doi:10.1371/journal.pone.0038024)
Supplement: Text S1 — RNA Synthesis and Purification. (DOCX) [file pone.0038024.s004.docx]

*RNA Synthesis and Purification*

The DNA genes for L1 ribozyme ligase and the P4-P6 domain of the *Tetrahymena* Group 1 intron ([1](#_ENREF_1), [2](#_ENREF_2)), ∆C209 ([3](#_ENREF_3)) were synthesized by recursive PCR using commercially obtained oligonucleotides (Eurofins MWG Operon) designed to include a T7 promoter site, EcoRI and HindIII cleavage sites, and an end stability sequence. PCR products were confirmed by sequencing (Eurofins MWG Operon).

For L1 ligase, the four recursive PCR oligonucleotides were:

(1) 5'-TTCTAATACGACTCACTATAGGACTTCGGTCCAGTGCTCGTGCACTAGG-3'

(2) 5'-TTGCCGCTGGCAGCGGACCCACATGGTCGAACGGCCTAGTGCACGAGCACTGGAC-3'

(3) 5'-CGCTGCCAGCGGCAATCTGGCATGCTATGCGGAACCTTCACATCTTAGACAGGAG-3'

(4) 5'-GCGACTGGACATCACGAGGCACCTAACCTCCTGTCTAAGATGTGAAGGTTCC-3'

The L1 ligase PCR primers were

(5) 5'-GTGGGAATTCTAATACGACTCACTATAGGACTTCGGTCC-3' (forward)

(6) 5'-CACCAAGCTTGCGACTGGACATCACGAGGCAC-3' (reverse)

For P4-P6 domain, the four recursive PCR oligonucleotides were:

(1) 5'-GTGGGAATTCTAATACGACTCACTATAGGGGAATTGGGGGAAAGGGGTCAACA GCCGTTC-3'

(2) 5'-ACCATACCCTTTGCAAGGCCTACTCAAAGTTTCCCCTGAGACTTGGTACTGAACGGCTGT

TGACCCCTTT - 3'

(3) 5'-GGCCTTCGAAAGGGTATGGTAATAAGCTGACGGACATGGTCCTACCCCCAGCCAAGTCC

TAAGTCAACA-3'

(4) 5' - CACCAAGCTTGAACTGCATCCATATCAACAGAAGATCTGTTGACTTAGGACTTGGCTG

GG - 3'

The P4-P6 domain PCR primers were

(5) 5'-GTGGGAATTCTAATACGACTCACTATAGG-3' (forward)

(6) 5'-CCTATAGTGAGTCGTATTAGAATTCCCAC-3' (reverse)

Aliquots of completed PCR reactions were purified by electrophoresis in agarose. Bands at the expected sizes were excised from gels and recovered (DNA Clean and Concentrator Kit, Zymo Research ). The purified PCR products were ligated into pUC19 plasmid (T4 Quick Ligation Kit, New England Biolabs). Competent *E*. *coli* dH5α cells were transformed with the ligation products. Clonal cell lines were established from transformations and used to produce bulk quantities of plasmid constructs for use as templates in transcription reactions (Endo Free Plasmid Maxi Kit, Qiagen).

The RNAs were produced by runoff transcription ([4](#_ENREF_4)) (MegaScript T7 Transcription Kit, Ambion), recovered by ammonium acetate precipitation, and resuspended in nuclease-free water (IDT). The product RNAs appear as tight bands when stained with Cybergold (Invitrogen) after electrophoresis in 8% denaturing polyacrylamide gels.

**References**

1. Prodromou C, Pearl LH (1992) Recursive PCR - a Novel Technique for Total Gene Synthesis. *Protein Eng* 5:827-829.

2. Sandhu GS, Aleff RA, Kline BC (1992) Dual Asymmetric PCR - One-Step Construction of Synthetic Genes. *BioTechniques* 12:14-16.

3. Juneau K, Podell E, Harrington DJ, Cech TR (2001) Structural Basis of the Enhanced Stability of a Mutant Ribozyme Domain and a Detailed View of RNA--Solvent Interactions. *Structure* 9:221-231.

4. Sampson JR, Uhlenbeck OC (1988) Biochemical and Physical Characterization of an Unmodified Yeast Phenylalanine Transfer RNA Transcribed in Vitro. *Proc Natl Acad Sci U S A* 85:1033-1037.
